# Prevalence and Predictors of Obesity, Undernutrition, and Anemia in Women of Reproductive Age Living in Nepal: A Scoping Review

**DOI:** 10.1155/jnme/8897249

**Published:** 2025-10-28

**Authors:** Shishir Paudel, Tulsi Ram Bhandari, Yoko Oda Thapa, Amar Nagila, Anisha Chalise, Nawaraj Chapagain, Sakai Hiroko

**Affiliations:** ^1^Kathmandu Institute of Child Health, Hepali Height, Budhanilkhantha, Kathmandu, Nepal; ^2^School of Health and Allied Sciences, Faculty of Health Science, Pokhara University, Pokhara, Nepal; ^3^Nutrition Research Project of Kansai Medical University Japan in Annapurna Rural Municipality, Kaski, Nepal; ^4^Center for Research on Environment, Health and Population Activities (CREHPA), Lalitpur, Nepal; ^5^Faculty of Nursing, Graduate School of Nursing, Kansai Medical University, Hirakata, Japan

**Keywords:** anemia, malnutrition, obesity, overweight, women of reproductive age

## Abstract

Malnutrition remains a critical public health issue among women of reproductive age (WRA) worldwide, and Nepal is no exception, as the country experiences a triple burden of malnutrition among WRA, including undernutrition, overnutrition, and anemia. This scoping review aimed to map and synthesize the existing literature to explore the scope of research on the prevalence and predictors of malnutrition (underweight, obesity, and anemia) among WRA in Nepal. A comprehensive search was conducted using several databases, including MEDLINE, DOAJ, CINAHL, and NepJOL, covering studies published between January 1, 2000, and June 15, 2024. Studies reporting the prevalence and/or predictors of undernutrition, obesity, or anemia among women aged 15–49 years in Nepal were included in this review. The exclusion criteria were studies focusing on disease-specific malnutrition, review articles, and clinical trials. Studies relying solely on secondary analyses of Nepal Demographic and Health Survey (NDHS) data were excluded to prevent duplication of estimates. A total of 1448 records were retrieved, and 751 duplicates were removed, leaving 697 records for screening. After excluding 577 records at the title/abstract stage, 120 full texts (including 6 from citation searching) were assessed, and 16 studies met the inclusion criteria. The prevalence of underweight ranged from 2.0% to 30.3%; overweight/obesity ranged from 4.8% to 55.0%; and anemia ranged from 12.83% to 66.8%. Definitions and measurement methods varied considerably across studies, contributing to wide variability in reported prevalence estimates. The studies identified numerous factors associated with malnutrition. Demographic and socioeconomic characteristics such as age, ethnicity, family size, educational status, occupation, food consumption patterns, and health-seeking behavior were linked to different forms of malnutrition. Anemia was associated with women's ethnicity, deworming medication, and reproductive health factors, such as menarcheal status and number of antenatal visits. This review maps the existing research on malnutrition among WRA in Nepal, identifying key trends and critical gaps that require further investigation. It also emphasizes the need for multisector-targeted interventions to address the diverse factors contributing to malnutrition, including anemia.

## 1. Introduction

Malnutrition, in its various forms, including undernutrition, overweight, obesity, and micronutrient deficiencies, remains a significant public health concern worldwide [1]. Malnutrition impacts people of all ages. Globally, in 2022, an estimated 43% of adults aged > 18 years were overweight, while 16% were living with obesity [2]. In the same year, an estimated 2.5 billion adults were overweight, including 890 million with obesity, while 390 million children were underweight [2]. Malnutrition is of particular concern among women of reproductive age (WRA), as global estimates suggest that women are affected by the triple threats of anemia, underweight, and overweight. Globally, 30% of women aged 15–49 years are estimated to live with the debilitating effects of anemia, with the highest prevalence observed among women living in South Asia (49%) [3]. Similarly, the burden of underweight among women is higher in South Asia, with 22% of women aged 20–49 years estimated to be underweight, compared to the global average of 10%. In terms of overweight and obesity, 35% of women aged 20–49 years are estimated to be overweight, with 13% living with obesity [3]. Nepal is no exception to this global health crisis.

Nepal is a landlocked country in South Asia, bordered by China to the north and India to the south, east, and west. It is characterized by diverse topography, ranging from the lowland Terai plains to the high Himalayan mountains. The country has significant variations in socioeconomic conditions, food security, and healthcare access, which influence the nutritional status of its population, including WRA [4, 5]. WRA constitutes a significant portion of Nepal's population, comprising approximately 28.22% of the national population [6]. This demographic is particularly vulnerable to early marriage and adolescent pregnancy [7]. The country faces a double burden of malnutrition, with both undernutrition and overnutrition coexisting. However, with rising micronutrient deficiencies, triple burden of malnutrition has also been noted, with increasing anemia and overweight/obesity rates in WRA. The Nepal Demographic Health Survey (NDHS) estimates for 2022 suggest that almost 26% of adolescent girls (15–19 years) were thin, while 6% were overweight/obese. Among WRA (20–49 years), an estimated one in ten women was underweight (10%), while the overweight and obesity rates were 26% and 8%, respectively. Similarly, the estimates suggest that the prevalence of anemia among women aged 15–49 years in Nepal increased from 36% in 2006 to 41% in 2016 and declined to 34% in 2022 [8].

A complex interplay of factors, such as lower socioeconomic status, gender inequalities, inadequate diet and/or dietary diversity, food insecurity, poor health, and behavioral and consumption patterns, contributes to malnutrition in WRA [9, 10]. Early marriage, high parity, and short birth intervals can also deplete a woman's nutritional reserves and contribute to undernutrition [2, 11]. In addition to these socioeconomic and demographic factors, environmental contaminants, autoimmunity, and nutrient malabsorption can contribute to anemia [12]. The consequences of malnutrition in WRA are far-reaching, as undernutrition could increase the risk of anemia, weaken the immune system, and lead to adverse pregnancy outcomes [13]. Overweight/obesity, on the other hand, could lead to noncommunicable diseases, such as cardiovascular disease, diabetes, and certain cancers. Anemia increases the risk of cognitive and physical developmental impairments, especially in vulnerable populations, and may also lead to increased mortality and morbidity [2, 14]. Malnutrition in WRA also has intergenerational effects, as it can lead to the continuation of the cycle of malnutrition through the birth of low-birth-weight infants, and malnourishment in children has been linked to multiple health problems in the mother and her fetus [15, 16].

Nepal has made significant commitments and efforts to address malnutrition, including in WRA. In 2011, Nepal joined the Scaling Up Nutrition Movement, a global initiative to improve nutrition, uniting multiple sectors and agencies for a collective effort to improve nutrition [17]. Nepal's Multi-Sectoral Nutrition Plan II (MSNP II, 2018–2022) engaged multiple government sectors to reduce stunting and undernutrition in WRA. The plan aimed to lower the prevalence of undernutrition (body mass index [BMI] < 18.5) in WRA from 17% to 12% by 2022. Similarly, it also aimed to lower the prevalence of overweight and obesity among WRA to 12% and reduce the percentage of anemia among adolescent girls (10–19 years) to 25% and among WRA (15–49 years) to 24% by 2022 [18]. Regardless of these multiple efforts, the challenge of malnutrition among WRA persists in Nepal.

Understanding the prevalence and predictors of these nutritional conditions is crucial for developing targeted interventions to address the nutritional needs and well-being of children and WRA and improve maternal and child health outcomes in developing nations, including Nepal. However, comprehensive reviews addressing the prevalence and predictors of obesity, underweight, and anemia among WRA in Nepal are lacking. Given the variability in study populations and methodologies, a scoping review approach is appropriate to map the extent and nature of research in this area and to identify existing gaps that require further investigation. This review aims to synthesize current knowledge on the prevalence and predictors of malnutrition among WRA in Nepal, providing insights into trends and research gaps that can inform future studies.

## 2. Materials and Methods

The scoping review followed Arksey and O'Malley's framework [19], including six key steps: identifying research questions; identifying relevant studies; selecting studies to be included in the review; charting the data and quality appraisal; collating, summarizing, and reporting the results; and consulting stakeholders.

### 2.1. Identifying the Research Question

The main research question was “What is known about the prevalence and predictors of malnutrition among WRA in Nepal and what gaps exist in the literature?” This research question aligns with the Population, concept, and context (PCC) framework, with the population of interest defined as WRA (15–49 years) by the World Health Organization (WHO). This review aims to identify the volume and nature of existing studies on malnutrition among WRA in Nepal, map trends, and highlight key gaps to inform future research. The review also aimed to map the extent of the problem and identify key factors contributing to malnutrition in this population. This review focused on Nepal, considering the country's unique cultural, geographical, and socioeconomic dynamics, including the disparities between rural and urban populations. This comprehensive approach provides valuable insights into the factors influencing malnutrition among WRA in Nepal and helps identify gaps in the existing literature, paving the way for future research.

### 2.2. Data Sources and Search Strategy

The literature search was initially limited to the bibliographic databases: MEDLINE, Directory of Open Access Journals (DOAJ), CINAHL, and NepJOL (as many Nepalese journals are indexed in NepJOL). The search timeline was from 1^st^ January 2000 to 15^th^ June 2024, reporting on the nutritional status of WRA in Nepal. Additional articles were identified through the reference lists of the retrieved articles and the Google Scholar search engine. A comprehensive search strategy was developed using search terms identified from the published literature, along with some Medical Subject Headings (MeSH) terms, such as “Nutritional Disorder” OR “Nutritional Status” OR “Malnutrition” OR “Malnourishment” OR “Anemia” OR “Overnutrition” OR “Overweight” OR “Predictors” AND “Prevalence [Abstract]” AND “Nepal.” The search strategy also incorporated terms used to identify WRA in Nepal. The complete database-specific search strategies, including Boolean strings, filters, and exact dates of search, are provided in Supporting File 1. This review adhered to the preferred reporting items for systematic reviews and meta-analyses extension for scoping reviews (PRISMA-ScR) checklist [20]. The protocol for this review was registered under PROSPERO [CRD42024512066] as a systematic review. However, during the review process, it became clear that the available studies involved diverse populations and adopted varying methodologies to assess malnutrition. Due to this heterogeneity, a traditional systematic review focusing on pooled quantitative synthesis was not feasible. Therefore, the review was adapted to a scoping review, which is better suited to map the breadth of evidence, summarize methodological approaches, and identify research gaps.

### 2.3. Study Selection

The inclusion criteria were as follows: studies reporting the prevalence of malnutrition (underweight, overweight, obesity, and anemia) and its risk factors among WRA in Nepal published between 1^st^ January 2000 and 15^th^ June 2024. Studies were excluded on the basis of several criteria. First, those that did not clearly define or report on undernutrition, obesity, or anemia were excluded. Studies focusing on malnutrition caused by specific diseases or clinical conditions were also excluded. Additionally, review articles, clinical trials, studies relying solely on secondary data analyses (such as those based on the Nepal Demographic and Health Survey [NDHS]), and studies published in languages other than English were excluded. NDHS-based secondary analyses were not included in the review process to prevent duplication of findings, as multiple papers often use the same NDHS dataset, which would have led to repeated estimates and observations within the review. Initially, the researcher (SP) screened the titles and abstracts of the retrieved articles, focusing on the nutritional status of WRA. After title and abstract reviews, the full texts of potentially eligible articles were acquired.

### 2.4. Charting the Data and Quality Evaluation of Articles

Two researchers (SPand AC) jointly compiled the list of full-text articles and independently extracted data from the included studies. A total of 16 studies were included in this review. The extracted data included study characteristics, including the author's name, country of origin, years of publication, the aim of the study, and key methodologies such as study design, study setting, sample size, and age of the population, along with outcome measures including prevalence rates of malnutrition indicators and significant risk factors. The data were charted using a structured data extraction form using Microsoft Excel. The senior researcher (SH) independently performed an additional search for subscription-based articles and provided another charted Excel file that was merged with the original Excel sheet.

Quality appraisal was performed after the selection of the 16 studies to ensure a structured and systematic evaluation of the study's credibility and relevance. Two independent researchers (TRB and SP) determined the quality of the included studies. The included articles were read extensively, and the critical appraisal tool provided by Loney et al. [21] was adapted for the critical appraisal of health research literature based on the prevalence or incidence of health problems. Articles scored one point for each of the following quality markers: (i) the study design and sampling method were appropriate for the research question; (ii) the study had an appropriate and unbiased sampling frame; (iii) the study had an adequate sample size; (iv) the study used objective, suitable, and standard criteria to measure nutritional status in the sample population; (v) unbiased assessors measured nutritional status in the study; (vi) the study had adequate responses (> 70%) or participant refusal was described; (vii) the study provided confidence intervals (CIs) of estimated prevalence; and (viii) the study participants and setting were described in detail [21]. Studies with scores of < 3 were excluded in the second stage to maintain the quality of the evidence synthesized.

### 2.5. Extraction and Synthesis

From the included publications, the study area, participants' demographic information, sample size, duration of the study, applied diagnostic instruments for malnutrition, the observed prevalence of different nutritional conditions, hypothesized risk factors, and factors associated with nutritional conditions were extracted by researchers (SP, AC, and NC). Data extracted from the full texts of the included articles were read repeatedly to familiarize with the data collected, and the extracted data were arranged into tables based on the appropriate themes. While our primary focus was WRA (15–49 years) as defined by WHO, the search process revealed there is limited research in Nepal specifically targeting this exact age group. Therefore, to comprehensively capture the landscape of malnutrition among WRA, we also included studies with slightly broader age ranges (e.g., 18–64 years) and those focused exclusively on adolescents (10–19 years). These studies were included only if they provided relevant data for women and adolescent girls within or adjacent to the WRA range. Unfortunately, we did not have access to individual-level raw data to isolate the 15–49 subset. Consequently, no subset extraction or sensitivity analyses could be conducted. The authors (HS, TRB, and SP) held regular meetings to discuss the results. Any disagreements during the screening and data extraction phases were resolved through discussions with the co-authors.

### 2.6. Consultation

To ensure the rigor and reliability of the findings, the senior researchers (TRB and SH) independently consulted experts with experience in systematic reviews, scoping reviews, and nutrition in the Nepalese context. The research team discussed and finalized the synthesis and overall review. The expert consultation provided critical insights into structuring the review, particularly in addressing the methodological heterogeneity of the included studies, which encompassed community-based, hospital-based, and school-based designs, as well as diverse indicators such as BMI, anemia, and midupper arm circumference (MUAC). Given these variations, a scoping review was reaffirmed as the appropriate approach, allowing for a broad synthesis of findings while effectively mapping existing literature. Additionally, discussions highlighted the limited research available on the nutritional status of WRA in Nepal, particularly in relation to regional disparities and socioeconomic influences. These insights were instrumental in shaping the study's scope, ensuring it effectively identified research gaps and provided a foundation for future investigations.

## 3. Results

### 3.1. Search Outcomes

**Figure 1:**
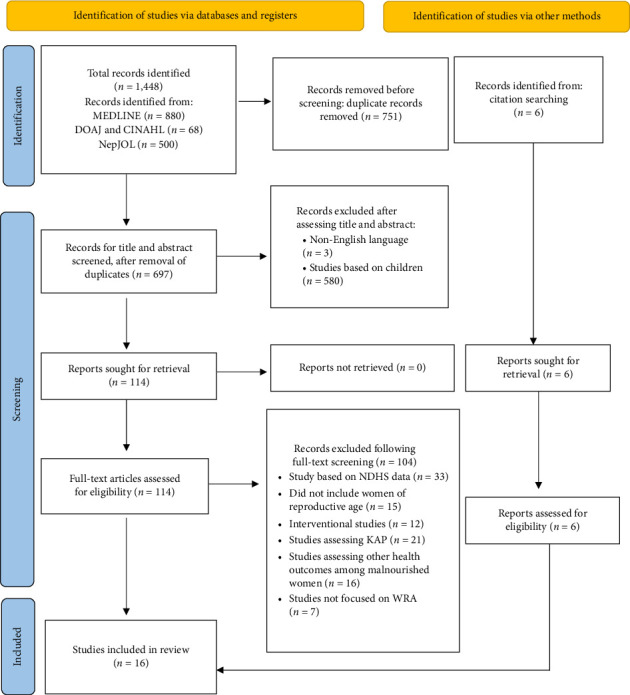
PRISMA flow diagram of the study selection.

A total of 1448 records were retrieved. After the removal of 751 duplicates, 697 records remained for screening. Of these, 577 were excluded at the title/abstract stage, and 120 full texts (including 6 identified through citation searching) were assessed for eligibility. Ultimately, 16 studies met the inclusion criteria (Figure 1).

### 3.2. Study Characteristics and Quality Appraisal

Sixteen studies were included in this review, examining various aspects of malnutrition among WRA in Nepal. The studies varied in their designs, sample sizes, and geographical coverage, providing a comprehensive overview of nutritional status and its determinants in this population. Among the 16 studies, 7 were recently published after 2020, nine were published between 2013 and 2020, and none were published before 2013. Fifteen studies employed a cross-sectional design, while one utilized secondary data from a larger interventional study. Most of the studies (nine) were community-based, while four were hospital-based studies, two were school-based studies, and one was a secondary analysis. Nine studies explicitly targeted WRA (15–49 years), while two studies focused solely on adolescent girls aged 10–19 and 15–19 years, respectively, while two others included pregnant women aged 16–50 years and 18–37 years. Additionally, three studies reported on women with broader age ranges as follows: 18–64 years and 20–59 years.

Quality appraisal of the included studies was conducted using the critical appraisal tool provided by Loney et al. [21] in which the selected studies received a minimum score of three and a maximum score of seven. The average quality score was 5.2; notably, eight studies received a score of ≤ 5. Several studies had methodological limitations, including missing or inconsistently reported CIs (*n* = 14), unclear assessor credibility (*n* = 12), small sample sizes (*n* = 7), and unclear sampling techniques (*n* = 6). Overall, the quality appraisal indicated that the findings from these studies were of moderate quality yet crucial to provide valuable insights into the malnutrition status of WRA in Nepal (Table 1). However, these limitations also highlight the need for cautious interpretation of the reported prevalence and associated factors.

### 3.3. Prevalence of Malnutrition

The prevalence of different forms of malnutrition among WRA varied across studies. Ten studies assessed nutritional status using BMI, three assessed anemia, and two assessed BMI and anemia [23, 35]. One study assessed acute malnutrition through MUAC readings [27]. Considerable variability was observed across studies in terms of geographic region, age range of participants, and diagnostic criteria. For instance, among the 12 studies assessing nutritional status using BMI, the categorization varied across studies, but most generally identified underweight, normal weight, overweight, and obese statuses. However, few studies merged underweight with normal weight [29, 33] and/or overweight with obesity [9, 29, 35], which limited the assessment of the true proportion of these attributes. Given the heterogeneity in study designs and methods, these findings provide a broad overview of existing research rather than a pooled estimate.

The prevalence of underweight in the selected studies ranged from 2.0% to 30.3%. Higher rates were reported in the hilly region with a wide range of 2.7%–32.3%. Studies from the mountain region also indicated a concerning underweight prevalence, ranging from 24.7% to 30.48% [23, 24]. The prevalence of underweight in the Terai region ranged from 2% to 31% [25, 32]. Similarly, the rate of overweight/obesity was found to be between 4.8% and 55.0%. Higher rates were observed in the hilly region, with a broad prevalence range of 4.8%–49.6%. In the Terai region, the overweight and obesity rates ranged from 26.7% to 32.7%. The mountain region reported a prevalence rate of 4.3%–28.6%. Likewise, in the case of anemia, the assessments from four studies revealed diverse outcomes, with anemia being prevalent among pregnant women (17.9%–66.8%) [30, 33], while 12.8% of WRA (20–49 years) [23] and 51.3% of adolescent girls (10–19 years) had anemia [34]. The highest rates of anemia were reported in the Terai region (66.8%) [28], followed by the hilly (51.3%) [34] and mountain regions (12.85%–17.9%) [23, 29]. Similarly, few studies assessed the MUAC and waist circumference (WC), indicating acute malnutrition in 9.9% of female adolescents aged 15–19 years, while abdominal obesity was observed in 89.8% of WRA (15–49 years) [27, 33] (Table 2).

### 3.4. Factors Associated With Malnutrition

Sixteen studies were included to review the factors associated with malnutrition, while two were excluded because they were descriptive studies and one lacked precision in the analysis. A comprehensive review of studies that examined the factors associated with BMI and anemia among diverse populations yielded significant insights into the hypothesized and confirmed determinants of these conditions. The risk factors of malnutrition, including underweight, overweight, obesity, and anemia, revealed a complex interplay between demographic, socioeconomic, and lifestyle variables.

For BMI, age was hypothesized to be an associated factor in eight studies (Table 3), of which six found it to be associated. Regarding WRA aged 35–49 years, those aged 25–34 years had 1.3 times higher odds (adjusted odds ratio [aOR]: 1.3, 95% CI: 1.2–1.4) and those aged 15–24 years had twice the odds (aOR: 27, 95% CI: 2.5–3.0) of experiencing malnourishment, suggesting decreasing odds of malnutrition with increasing age [35]. In contrast, Tripathi et al. suggested higher odds of overweight/obesity with increasing age [29]. Educational status was another frequently hypothesized determinant of undernourishment and overweight and was consistently found to be significant across multiple studies. Similarly, socioeconomic factors, such as occupation, monthly income, and household food insecurity, were also highlighted by some of the studies as important predictors of nutritional status. Tripathi et al. suggested that those engaged in business had a seven-fold increase in the odds of overweight/obesity compared with those unemployed (aOR: 7.37, 95% CI: 2.25–14.17) [29]. Similarly, household characteristics, including family size, parental education, and food consumption patterns, were also significantly associated with underweight, overweight, and obesity in multiple studies. Aryal et al. suggested that, compared with those consuming > 5 food groups in their diet, individuals consuming ≤ 4 food groups have a tenfold (aOR: 10.06, 95% CI: 3.35–30.24) increase in the odds of acute malnourishment (MUAC < 22 cm) [27].

Regarding anemia, four of the studies hypothesized age to be associated with an anemic condition, while none of them established a significant relationship between age and anemia. Sharma et al. reported ethnicity as an important factor associated with anemia among pregnant women, as those in the disadvantaged Janajati communities had four times higher odds of having anemia (aOR: 4.615, 95% CI: 1.48–14.35) [30]. A similar observation was made by Yadav et al., who noted that pregnant women belonging to Terai Dalit/Janajati/Muslim communities had twice (aOR: 2.34, 95% CI: 1.06–5.7) the odds of having anemia compared with those belonging to the Madhesi community [28]. Compared with individuals who consumed deworming medicines, those who did not consume deworming medicines had thrice the odds of experiencing anemia (aOR: 3.03; 95% CI: 1.20–7.65) [28]. Furthermore, reproductive health factors, such as menarcheal status and number of antenatal visits, were significantly associated with anemia (Table 3) [28, 34].

The collective synthesis of these selected studies suggests that younger women were more likely to be underweight, while those in older reproductive ages had increased odds of overweight and obesity. Socioeconomic disadvantage, including low education, unemployment, and food insecurity, was consistently associated with malnutrition, whereas higher education was protective. Poor dietary diversity, low protein intake, high fat consumption, and alcohol use contributed to both undernutrition and obesity. Reproductive factors such as early pregnancy, high parity, and limited antenatal care increased the risks of malnutrition and anemia. Health-related conditions, including lack of deworming and worm infestation, further heightened vulnerability to anemia (Table 4).

## 4. Discussion

This review revealed significant variations in the prevalence of different forms of malnutrition among WRA in Nepal; however, all findings revealed a high burden of malnutrition. The prevalence of underweight ranged widely, from 2.0% to 30.3%, while that of overweight/obesity ranged from 4.8% to 55.0%. The hilly region was shown to experience double the burden of undernutrition and overnutrition, but a wider variation exists in the prevalence rates across the studies. However, in the case of the mountain region, the prevalence of underweight was high and almost constant across studies [23, 24], revealing more concerning issues of undernutrition in the hilly region. Similarly, overweight and obesity were observed as a public health issue in the Terai region [25, 32] However, variations in the age of the study populations, demographic characteristics, and the approach used to categorize BMI scores among the selected studies made aggregating a pooled prevalence of malnutrition among WRA in Nepal challenging. Nevertheless, the findings provide a magnitude of the issue and highlight the widespread nature of malnutrition among WRA in Nepal. The findings suggest that the burden of malnutrition in Nepal is higher than the estimates from the NDHS report of 2022, as the NDHS suggests that only 10% of WRA were estimated to be thin/underweight, while 6% of adolescent girls and 8% of WRA were estimated to be overweight/obese [8]. Several factors could account for these discrepancies. For instance, some of the reviewed studies included pregnant women in their samples, which may have influenced reported prevalence rates. Similarly, differences in study settings may have influenced the results, as NDHS is a nationally representative survey, while many of the reviewed studies focused on specific regions, some of which may have higher malnutrition rates. By excluding NDHS-based secondary analyses, we focused on mapping unique, primary datasets while using NDHS only as a benchmark for comparison.

The observed prevalence of underweight, overweight, and obesity among WRA in Nepal aligns closely with global and regional estimates [37–40]. For instance, a study based on secondary analysis of Demographic and Health Surveys (DHS) from 52 low- and middle-income countries (LMICs) revealed that 15.2% (95% CI: 15.1–15.3) of WRA were underweight, while 19.0% (95% CI: 18.9–19.1) were overweight and 9.1% (95% CI: 9.0–9.2) were obese [41]. Similarly, a systematic review and meta-analysis based on 112 studies from Asia, suggested a pooled prevalence of underweight of 28% (95% CI: 25%, 31%) and overweight of 17% (95% CI: 15%, 19%) in South Asia, while the prevalence rates were 20% (95% CI: 15%, 26%) and 20% (95% CI: 15%, 24%), respectively, in Southeast Asia [42].

During the review, anemia was noted to be a significant concern among WRA in Nepal, affecting 12.8% of WRA (20–49 years) [23] and 51.3% of adolescent girls [34]. While the primary focus was on nonpregnant WRA, two studies included pregnant women. These were retained to provide a broader perspective on maternal nutrition, given the strong links between pregnancy and nutritional outcomes. However, findings specific to pregnant women were interpreted separately to avoid biasing the overall conclusions. Pregnant women had the highest anemia prevalence, ranging from 17.9% to 66.8% [30, 33]. These findings are consistent with the findings from the NDHS results 2022, in which 34% of women aged 15–49 were found to have anemia [8]. Global estimates based on 46 DHS reports from LMICs from 2010 to 2021 also reflect similar trends, with anemia affecting 45.2% (95% CI 41.2–49.2) of pregnant women and 39.5% (95% CI 33.9–45.2) of nonpregnant women [43]. Similarly, another analysis based on 133 countries between 2000 and 2019 indicated that the global prevalence of anemia in nonpregnant women aged 15–49 years remained almost stable at 30% in 2019 from 31% in 2000, while it decreased from 41% to 36% in pregnant women aged 15–49 years [44]. The WHO has set a target to reduce the prevalence of anemia among WRA to ≤ 15.2% by 2025 [45]. Based on these findings, Nepal has a long way to go to meet this critical public health goal.

The wide range of prevalence estimates observed in this review reflects substantial heterogeneity among the included studies. Geographic variation was notable, with studies from the mountain region often reporting higher underweight prevalence, while overweight and obesity were more common in the Terai. Differences in age ranges further contributed to variability, as some studies included adolescents or pregnant women in addition to nonpregnant adults. Finally, the variations in outcome definitions, such as different BMI cut-offs or methods of anemia assessment, directly influenced reported prevalence values. These factors collectively underscore why direct statistical pooling was not feasible and why results are best interpreted within their specific study contexts.

### 4.1. Predictors of Malnutrition

Undernutrition among WRA in Nepal was associated with multiple demographic and socioeconomic factors. The reviewed studies suggested a complex relationship between age and malnutrition. While some studies have indicated higher odds of undernutrition among younger WRA (15–24 years) compared with older age groups (35–49 years), others have reported a positive association between age and overweight/obesity. This complex relationship between age and nutritional status has been observed in previous studies, in which increasing age was found to be positively associated with being overweight, and obesity had a positive association with age and was negatively associated with undernutrition [37, 41, 42, 46]. Education level was significantly associated with nutritional outcomes in Nepal. Women with higher educational attainment were less likely to experience malnutrition, including anemia. A systematic review and meta-analysis based on women in South and southeast Asia suggested that the pooled prevalence of underweight was two times higher among South Asian women with no formal education compared with those with secondary and higher education [42]. Similarly, a study based on DHS findings from 46 LMICs found higher odds of anemia among both pregnant mothers (aOR = 1.75; 95% CI 1.63–1.89) and nonpregnant women (aOR = 1.23; 95% CI 1.20–1.25) with no formal education when compared with those with higher education [43]. The interconnection between educational and nutritional statuses has been shared by multiple studies worldwide [41]. The reason might be that education empowers women with knowledge of nutrition and health practices, enabling them to make informed decisions.

Studies in Nepal have indicated that women from lower socioeconomic backgrounds and those facing food insecurity are more likely to experience undernutrition and anemia. This finding aligns with global research in which socioeconomic status is consistently associated with nutritional outcomes [41, 43, 46]. A systematic review suggested that the prevalence of underweight in South Asia was more than three times higher in the poorest households than in the richest households, where the prevalence of overweight was six times higher in the richest households than in the poorest households [42]. This highlights the role of household wealth as a major driver of malnutrition worldwide, affecting dietary quality and access to health services. Household wealth is also connected with dietary habits, which have emerged as a crucial determinant of nutritional status among WRA in Nepal. Aryal et al. observed a tenfold increase in the odds of undernutrition among women consuming fewer than five food groups compared with those with a more diverse diet [27]. Globally, dietary diversity is recognized as a key factor in preventing malnutrition [1, 42]. Furthermore, climate change and the increasing frequency and magnitude of extreme climatic events in Nepal have been linked to poor food production and food insecurity, exacerbating the rising trend of malnutrition in the country and affecting mostly the vulnerable population [4]. A systematic review conducted in sub-Saharan African countries reported that high protein and sugar intake increased the risk of overweight and obesity [47]. The similarity in findings emphasizes dietary diversification as a key strategy to combating malnutrition among WRA.

### 4.2. Gap in the Literature

This review also revealed significant gaps in the literature regarding the factors affecting the nutritional status of WRA in Nepal. Several globally recognized determinants of women's nutritional status in LMICs, such as cultural and religious practices, women's autonomy and household decision-making, and gender roles [48, 49], were not assessed in the reviewed studies. Given that gender norms and social structures play a crucial role in influencing dietary behaviors and healthcare access, future research should examine how these sociocultural dimensions impact nutritional status among WRA. Additionally, the roles of healthcare access, health literacy or nutritional literacy, maternal workload, and exposure to any nutritional intervention, which are crucial to understanding and addressing malnutrition [50–52], have been largely overlooked. Addressing these gaps in future research could provide a more comprehensive understanding of the factors influencing malnutrition and inform targeted intervention strategies. Understanding how these factors shape dietary behaviors and nutrition-related decision-making could provide critical insights for designing effective interventions. Another significant gap is the limited exploration of regional disparities in malnutrition. While some studies report variations in undernutrition, overweight, and anemia across Nepal's mountain, hill, and Terai regions, systematic analyses of the underlying determinants are lacking. Regional differences in food availability, agricultural practices, healthcare infrastructure, and socioeconomic conditions may contribute to the observed disparities, yet these factors remain insufficiently studied. Future research should adopt a geographically stratified approach to better understand the interplay between regional determinants and nutritional outcomes, which could inform more targeted interventions. In addition, while the relationship between education and nutritional outcomes has been explored, there is a lack of research on the role of health literacy and the effectiveness of past nutrition interventions in Nepal. Various government and nongovernmental programs, such as food fortification, micronutrient supplementation, and dietary education campaigns, have been implemented to combat malnutrition. However, few studies have assessed their impact on dietary behaviors and health outcomes among WRA. Evaluating these interventions is essential to understand their effectiveness, identify gaps, and optimize future nutrition programs. Addressing these critical gaps in the literature will provide a more comprehensive understanding of the multifaceted determinants of malnutrition among WRA in Nepal. Future research should prioritize region-specific analyses and evaluations of health literacy and intervention programs. Strengthening the evidence base in these areas will be instrumental in designing effective, context-specific policies to combat malnutrition and improve the health of WRA in Nepal.

The findings of this scoping review have significant implications for public health policies and intervention programs aimed at addressing malnutrition in WRA in Nepal. Integrated strategies are urgently needed to address undernutrition and obesity among WRA in Nepal. Public health initiatives focus on improving access to nutritious foods, promoting healthy dietary practices, and providing education on the importance of balanced nutrition. Furthermore, future research should prioritize evaluating the effectiveness of interventions implemented in Nepal to address malnutrition among adolescents and WRA. These evaluations encompass a range of interventions, including nutritional education programs, food fortification initiatives, supplementary feeding schemes, and community-based interventions. Longitudinal studies are also crucial to track changes in nutrition indicators over time and assess the sustainability of intervention effects. In addition, programs aimed at reducing anemia, such as prioritizing iron supplementation and fortification efforts, are required, especially in regions with high prevalence rates.

Although this review provides valuable insights into the prevalence and burden of malnutrition among WRA in Nepal, certain limitations must be acknowledged. A key limitation was the scarcity of longitudinal studies, as we failed to find any longitudinal studies in this research area. Initially, this review was intended to be a systematic review and was registered with PROSPERO. However, owing to the diverse nature of the study populations and the varying methodologies adopted to quantify malnutrition in the included studies, deriving specific prevalence estimates or predictors was not possible. Consequently, a scoping review approach was adopted to better capture the breadth of existing research and identify the knowledge gap. In the future, once sufficiently high-quality, methodologically uniform studies are available, a systematic review could provide more precise estimates of the pooled prevalence and a comprehensive mapping of risk factors.

A notable limitation of this review was the inclusion of studies that did not strictly adhere to the WHO-defined reproductive age range of 15–49 years. Some studies included broader populations (e.g., 18–64 years), while others were limited to adolescent girls (10–19 years). These inclusions were necessary due to the scarcity of Nepal-specific studies focused exclusively on WRA. While this strategy allowed for a more comprehensive mapping of available evidence, it may have introduced heterogeneity in the prevalence estimates and associated factors. Unfortunately, individual-level data were unavailable, preventing us from extracting a 15–49-year subset or conducting sensitivity analyses to assess the robustness of our findings. Therefore, all results should be interpreted with caution, especially when generalizing to the 15–49 WRA population. Another limitation is the reliance on cross-sectional data, which limited our ability to track changes in prevalence and risk factors over time and establish causal relationships. The heterogeneity of the age groups of WRA considered in the studies is another limitation. This inconsistency makes direct comparisons between studies challenging and limits our ability to draw definitive conclusions regarding specific age ranges within the WRA population. A significant limitation of this review is that most of the studies had unclear assessor credibility, which raises concerns about the accuracy of data collection methods, while the small sample sizes from different studies potentially limit the generalizability of their findings. While these studies provided valuable insights into malnutrition, caution is needed when interpreting their results. Furthermore, the lack of complete data for all studies prevented us from conducting a meta-analysis. The absence of this approach limits the generalizability of the findings of this review. Addressing these gaps in future studies may enhance the effectiveness of intervention strategies and contribute to more equitable health outcomes.

## 5. Conclusions

This scoping review identifies a significant burden of malnutrition among WRA in Nepal and highlights gaps in the literature regarding social determinants, dietary patterns, and regional disparities. Future research should focus on addressing these gaps through longitudinal studies and targeted public health interventions. Although the specific prevalence varied across the studies, all pointed to a substantial public health concern. However, as several included studies were of moderate-to-low quality with missing CIs and unclear assessor credibility, these findings should be interpreted cautiously. Education, socioeconomic status, and dietary diversity are major determinants of malnutrition and anemia. These findings highlight the need for interventions that address both undernutrition and overnutrition, focusing on improving dietary practices, access to nutritious food, and targeted programs, such as health education interventions and supplementation with iron and other micronutrients, to tackle malnutrition and anemia among WRA.

## Figures and Tables

**Table 1:** Quality appraisal of selected studies.

Author (date)	Study design, setting, and population	Sample size and technique	Sampling frame	Outcome measures	Unbiased assessors	Response rate	Prevalence reporting	Score and limitations
Paudel et al. (2023) [22]	Community-based cross-sectional study, WRA (15–49 years)	426; Systematic random sampling	WRA residing at Pokhara Metropolitan	Height and weight measured	The assessor's credentials are not reported	Complete response	Prevalence of underweight overweight provided	Score 6: credibility of assessor unclear; CI of proportion not provided
Pokhrel B et al. (2023) [23]	Hospital-based cross-sectional study, WRA (20–49 years)	210; Complete enumeration	WRA attending OPD from June 1^st^ to 15^th^, 2018	Anemia test (Sahil's method [acid hematin method]), height and weight measured	The procedure for anemia test is described, but the assessor's credentials are not reported.	Complete response	Prevalence of mild and moderate anemia; underweight, preobesity, and obesity provided	Score 5: small sample size; credibility of assessor unclear; CI of proportion not provided
Giri et al. (2023) [24]	School-based cross-sectional study, Student (15–19 years)	400; quota sampling to cover students enrolled in classes 9–12	Female students from schools in Darchula district	Height and weight measured for BMI	The assessor's credentials are not reported	Data of 17 students excluded in bivariate analysis	Prevalence of thinness and overweight provided	Score 5: Nonrandom sampling; Credibility of assessor unclear; CI of proportion not provided
Divya et al. (2022) [25]	Community-based cross-sectional study, WRA (15–49 years)	339; systematic random sampling	789 household	Height and weight measured for BMI	The assessor's credentials are not reported	Complete response	Prevalence of undernourished, overweight, and obesity provided with 95% CI	Score 7: credibility of assessor unclear; CI of proportion not provided
Bhandari et al. (2022) [26]	Community-based cross-sectional study, women (15–49 years)	353; convenience sampling	Not mentioned	Height and weight measured for BMI	The assessor's credentials are not reported	Complete response	Prevalence of underweight, overweight, and obesity provided	Score 5: Nonrandom sampling; credibility of assessor unclear; CI of proportion not provided
Aryal et al. (2022) [27]	Hospital-based cross-sectional study, WRA (15–19 years)	452; purposive sampling	Women visiting antenatal clinics for 3 months	Adult MUAC tape	The assessor's credentials are not reported	444, missing sample unclear	Prevalence of acute malnutrition was provided	Score 4: nonrandom sampling; credibility of assessor unclear; missing sample; CI of proportion not provided
Yadav et al. (2021) [28]	Hospital-based cross-sectional study, pregnant women (18–37 years)	287, pregnant women attending antenatal care	Not applicable	Anemia test through laboratory test	The hospital lab technician performed a hemoglobin test (cyanmethemoglobin method)	Complete response	Prevalence of mild, moderate, and severe anemia provided with 95% CI	Score 7: small sample size and nonrandom sampling
Tripathi et al. (2020) [29]	Community-based cross-sectional study, WRA (15–49 years)	276; probability sampling but unclear	Not mentioned	Height and weight measured for BMI	Information on the data collection process and responsible personnel is missing	The initial sample was met	Prevalence of overweight/obesity provided	Score 4: unclear sampling; credibility of assessor unclear; CI of proportion not provided
Sharma et al. (2020) [30]	Hospital-based cross-sectional study, pregnant women (16–50 years)	319; unclear	Women visiting a hospital for delivery from Apr. to Sept. 2018	Hb photometer to assess Hb concentration and anemia	The procedure is mentioned but the assessor's credentials are not reported	Complete response	Prevalence of anemia provided	Score 4: unclear sampling; credibility of assessor unclear; CI of proportion not provided
Nepal et al. (2020) [31]	Community-based, cross-sectional study, women (18–64 years)	185; simple random sampling (lottery method)	Unclear	Height and weight measured for BMI	The assessor's credentials are not reported	Complete response	Prevalence of underweight, overweight, and obesity provided	Score 3: small sample size and unclear sampling; credibility of assessor unclear; CI of proportion not provided
Shah et al. (2020) [32]	Community-based, cross-sectional study, WRA (15–49 years)	144; simple random sampling but participant selection is unclear	Unclear	Height and weight measured for BMI	The assessor's credentials are not reported	144	Prevalence of underweight, overweight, and obesity provided	Score 3: Small sample size and unclear sampling; credibility of assessor unclear; CI of proportion not provided
Bhattarai et al. (2018) [33]	Community-based, cross-sectional study, women (15–49 years)	206; Probability sampling but participants selection is unclear	Unclear	Height and weight measured for BMI and Waist circumference	The assessor's credentials are not reported	Complete response	Prevalence of underweight, overweight, obesity, and abdominal obesity provided	Score 3: small sample size and unclear sampling; credibility of assessor unclear; CI of proportion not provided
Acharya (2017) [9]	Community-based, cross-sectional study, nonpregnant and nonlactating women (15–49 years)	229; unclear	Unclear	Height and weight measured for BMI	The assessor's credentials are not reported	Complete response	Prevalence of underweight and overweight/obesity provided	Score 3: small sample size and unclear sampling; credibility of assessor unclear; CI of proportion not provided
Kanodia et al. (2016) [34]	School-based cross-sectional study, adolescent girls aged 10–19 years	433; unclear if all school-going adolescent girls were enumerated	Unclear how many government schools were selected	Hemoglobin estimation using the cyanmethemoglobin method	Blood samples were sent to the hospital's central laboratory for Anemia tested	Complete response	Prevalence of anemia provided	Score 6: unclear sampling process; CI of proportion not provided
Bhandari et al. (2016) [35]	Secondary analysis of intervention study, WRA (15–49 years)	21,371; targeted nonrandom sampling (Secondary data)	Unclear	Height and weight measured. Blood samples were tested for by a laboratory technician using hematocrit machine	In health camps, doctors, nurses, and laboratory technicians check and assess participants' specific health conditions	The secondary analysis excluded 260 samples due to missing values	Prevalence of underweight and overweight/obesity provided	Score 4: prevalence of anemia is missing; few samples were excluded so possibility of bias; CI of proportion not provided
Shahi et al. (2013) [36]	Community-based, cross-sectional study, women (20–59 years)	110; Simple random sampling (Lottery method)	Unclear	Height and weight measured for BMI by PCL nursing 1st year students	Trained students collected data along with weight and height	Complete response	Prevalence of underweight, overweight, and obesity provided	Score 6: Small sample size and unclear sampling frame; CI of proportion not provided

**Table 2:** Prevalence of different nutritional indicators in past studies.

Author (date)	Population group	Operational definition of outcome variable	Prevalence of outcome variables
Paudel et al. (2023) [22]	WRA (15–49 years), hilly region	The BMI categories are not clearly mentioned	BMI: underweight = 13.6%; normal = 48.1%; overweight = 38.3%
Pokhrel et al. (2023) [23]	WRA (20–49 years), mountain region	Anemia is measured through Sahli's (acid hematin) method, where severe anemia was defined as Hb < 7 g/dL, moderate as Hb = 7–9.9 g/dL, and mild as Hb = 10–11.9 g/dL. For BMI, height was measured using a measuring tape and weight was measured using a portable weighing machine. A BMI cut-off point of < 18.5 kg/m^2^ suggested underweight, 18.50–24.99 kg/m^2^ suggested normal weight, 25.00–29.99 kg/m^2^ suggested preobesity, and > 30.00 kg/m^2^ suggested obesity	Anemia: mild = 23 (10.95%); moderate = 4 (1.90%); BMI: underweight = 64 (30.48%); normal = 86 (40.95%); preobese = 40 (19.05%); obese = 20 (9.52%)
Giri et al. (2023) [24]	Female students (15–19 years) mountain region	BMI < 18.5 kg/m^2^ was considered thinness, 18.5–24.9 kg/m^2^ as normal weight, 25–29.9 kg/m^2^ as overweight, and ≥ 30 kg/m^2^ as obese	BMI: thin (underweight) = 99 (24.7%); normal = 284 (71.0%); overweight = 17 (4.3%)
GC D et al. (2022) [25]	WRA (15–49 years), inner Terai region	WHO classification for BMI with BMI < 18.5 kg/m^2^ indicating underweight, 18.5–25 kg/m^2^ indicating normal, 25–30 kg/m^2^ indicating overweight, and ≥ 30 kg/m^2^ indicating obesity	BMI: undernourished = 105 (31.0%); overweight = 85 (25.1%, 95% CI: 20.5–30.0); obese = 26 (7.6%, 95% CI: 5.1–11.0)
Bhandari et al. (2022) [26]	WRA (15–49 years), hilly region	Weight and height were measured using electronic bathroom scales and standard stadiometers, respectively. BMI cut-off point of < 18.5 kg/m^2^ suggested underweight, 18.50–24.99 kg/m^2^ suggested normal BMI, 25.00–29.99 kg/m^2^ suggested overweight, and ≥ 30 kg/m^2^ suggested obese	BMI: underweight = 15 (4.0%); normal = 146 (41.0%); overweight = 173 (49.0%); obese = 19 (6.0%)
Aryal et al. (2022) [27]	WRA (15–19 years), Terai region	MUAC was used to assess the nutritional status as acutely malnourished (MUAC ≤ 21 cm) and not acutely malnourished (MUAC > 21 cm)	Acute malnutrition: MUAC ≤ 21 cm = 44 (9.9%)
Tripathi et al. (2020) [29]	WRA (15–49 years), hilly region	A BMI score < 25 kg/m^2^ was considered normal and a BMI score ≥ 25 kg/m^2^ was considered overweight/obese	BMI: normal/underweight = 139 (50.4%); overweight/obese = 137 (49.6%)
Nepal et al. (2020) [31]	Women (18–64 years), hilly region	The cut-off for BMI categories of underweight, normal, overweight, and obesity is unclear	BMI: underweight = 5 (2.7%); normal = 81 (43.8%); overweight = 69 (37.3%); obese = 30 (16.2%)
Shah et al. (2020) [32]	WRA (15–49 years), Terai region	Asian BMI cut-off was used to assess underweight, normal, and overweight/obese statuses	BMI: underweight = 3 (2.0%); normal = 60 (42.0%); overweight = 0 (0.0%); obese = 39 (26.7%)
Bhattarai et al. (2018) [33]	Women (15–49 years), hilly region	Weight was measured using a weighing scale and height using a stadiometer. Waist circumference (WC) was measured at the midpoint of the lower border of the rib cage and the iliac crest using a stretching tape. BMI was categorized as BMI < 25 = normal weight, BMI 25–30 = overweight, and BMI > 30 = obese. WC > 80 cm = abdominally obese and WC < 80 cm = normal	BMI: normal and underweight = 107 (49.5%); overweight = 66 (32.0%); obese = 33 (16.0%); abdominal obesity = 185 (89.8%)
Acharya et al. (2017) [9]	Nonpregnant/nonlactating women (15–49 years), hilly region	BMI was determined using the categories of nutritional status of adults reported by FAO (2005). BMI cut-offs < 18.5 kg/m^2^ were considered underweight, 18.5–24.9 kg/m^2^ as normal, 25–29.9 kg/m^2^ as overweight, and > 30 kg/m^2^ as obese	BMI: underweight = 74 (32.3%); normal = 144 (62.9%); overweight/obese = 11 (4.8%)
Kanodia et al. (2016) [34]	Adolescent girls (10–19 years), hilly region	Hemoglobin estimation was done by using the cyanmethemoglobin method. WHO recommended Hb < 12 g/dL was used to define anemia, as participants were adolescents	Anemia = 222 (51.3%) for adolescents aged 10–19, while 43.5% for adolescents aged 14–19
Bhandari et al. (2016) [35]	WRA covered districts from all three regions	The weights were measured on a digital scale and the heights were measured using a height scale. BMI < 18.5 was considered underweight (malnourished), 18.5 to 24.9 as normal, and ≥ 25 kg/m^2^ as overweight/obese. Anemia was defined as a hematocrit value less than 35% and normal as more than 35%	BMI: underweight = mountain (54 [6.0%]), hill (1161 [15.4%]), Terai (1235 [27.9%]); overweight/obese = mountain (217 [24.8%]), hill (1408 [18.7%]), Terai (332 [6.3%])
Shahi et al. (2013) [36]	Women (20–59 years), hilly region	BMI score was categorized based on the BMI for Asian index, where a BMI score < 18.4 was considered as underweight, 18.5–24.9 as normal, 25–29.9 as overweight, and BMI > 30 as obese	BMI: underweight = 30 (27.3%); normal = 51 (46.7%); overweight = 27 (24.5%); obese = 2 (1.8%)
Yadav et al. (2021) [28]^†^	Pregnant women (18–37 years), Terai region	Pregnant women were considered anemic if they had a hemoglobin concentration < 11.0 g/dL. Mild, moderate, and severe anemia were noted as Hb = 10.0–10.9 g/dL, Hb = 7.0–9.9 g/dL, and Hb < 7.0 g/dL, respectively	Anemia: mild = 186 (64.8% 95% CI: 59.0–70.3); moderate = 5 (1.7% 95% CI: 0.57–4.0); severe = 1 (0.3%)
Sharma et al. (2020) [30]^†^	Pregnant women, mountain region	Hb concentration was estimated using a Hb photometer (B hemoglobin, precision of 1 g/L, HemoCue AB, Sweden). The anemia cut-off value for pregnant women was < 12.3 g/dL	Anemia = 57 (17.9%)

^†^Studies based on pregnant women; outcome definitions (BMI standards, anemia assay cut-offs, including any altitude adjustment, MUAC thresholds, and pregnancy status) are provided as reported in the original studies.

**Table 3:** Factors associated with nutritional status among women of reproductive age.

Author (date); outcome measure	Hypothesized risk factors	Risk factors found to be significant
Paudel et al. (2023) [22]; normal BMI vs. abnormal BMI (underweight + overweight/obese)	Age (< 32 years, > 32 years); dependent members (Yes, No); education (illiterate and informal education and literate); occupation (own business and others); age of marriage (< 18, > 18); age at first pregnancy (< 20, > 20); parity (< 3, > 3); number of children (< 2, > 2); HFIS (food security, food insecurity)	Age (*p* < 0.001); dependent members (*p*=0.030); education (*p* < 0.001); occupation (*p*=0.002); age of marriage (*p* < 0.001); age at first pregnancy (*p*=0.006); parity (*p*=0.004); number of children (*p* < 0.001); FIS (*p*=0.017)
Pokhrel et al. (2023) [23]; (i) anemia: (anemia vs. nonanemia), (ii) BMI: (underweight vs. preobese vs. obese)	Anemia: age of the participants (15–19 years, 20–29 years, ≥ 30 years); BMI (underweight, normal, preobese, and obese). BMI: age (≤ 29 years, 30–39 years, 40–49 years); anemia (anemia and nonanemia)	Anemia vs. BMI categories (*p*=0.006)
Giri et al. [24]; low BMI (thin) vs. normal BMI	Age (15–16, 17–19 years); religion (Hindu, Christian); ethnicity (Dalit, Brahmin/Chettri); father's education (illiterate, informal education, primary level, lower secondary or secondary, and higher secondary and above); mother's education (illiterate, informal education, primary level, lower secondary or secondary, and higher secondary and above); monthly income of family (up to NRs. 2000, NRs. 2001 to 5000, and NRs. 5001 to 10,000); diversity on 24 h recall (< 5 food groups and ≥ 5 food groups); household food insecurity (food secure and food insecure); consume junk/processed food (Yes, No); missing meal (s) a day (No, Yes); initiation of mensuration cycle (Yes, No); food restriction during mensuration (Yes, No); worm infestation (Yes, No, Don't know); taken anthelminthic in last 6 months (Yes, No, Don't know); water treated (Yes, No, Don't know)	Age (*p*=0.016); father's occupational status (*p*=0.005); household food insecurity (*p*=0.002); kinds of food mostly brought in family (*p*=0.002); initiation of mensuration cycle (*p*=0.012); water treatment (*p*=0.028)
Bhandari et al. [26]; BMI ≤ 25 vs. BMI > 25	Age (15–25, 25–35, > 35 years); education (informal, primary, secondary, and higher); occupation (housewife, job, business, and student); marital status (unmarried and married); parity (none, 1 time, 2 times, > 2 times)	Age (*p* < 0.001); education (*p*=0.002); occupation (*p*=0.012); marital status (*p*=0.008); parity (< 0.001)
Aryal et al. (2022) [27]; acutely malnourished (MUAC ≤ 21 cm) vs. normal (MUAC > 21 cm)	Age (< 20 years, ≥ 20 years); gestational age (second trimester and third trimester); number of living children (≤ 2, ≥ 3); education (illiterate, literate); occupation (employed for cash/agriculture, unemployed/housemaker); husband's education (illiterate, literate); husband's occupation (employed for cash, employed not for cash/unemployed); annual household income (< national average, ≥ national average); kitchen garden in home (Yes, No); household food security (food secure, food insecure); dietary diversity (> 5 food groups, ≤ 4 food groups))	Education: literate vs. illiterate (aOR: 3.09, 95% CI: 1.43–6.70); occupation: employed for cash/agriculture vs. unemployed/housemaker (aOR: 3.16, 95% CI: 1.08–9.22); husband's occupation: employed for cash vs. employed not for cash/unemployed (aOR: 6.61, 95% CI: 2.17–20.12); dietary diversity: > 5 food groups vs. ≤ 4 food groups (aOR: 10.06, 95% CI: 3.35–30.27)
Yadav et al. (2021) [28]; anemic vs. nonanemic	Age (≤ 19, 20–24, 25–29, ≥ 30 years); ethnicity (Terai Dalit/Janajati/Muslim, Madhesi); educational status (< secondary level, ≥ secondary level); occupational status (homemaker and employed); pregnancy trimester (second and third); gravid (multigravida and primigravida); birth interval (≤ 2, > 2 years); history of miscarriage/abortion (Yes, No); No. of antenatal visits (< 4 times, ≥ 4 times); iron folic acid consumption (Yes, No); consumption of deworming medicine (Yes, No); food avoidance during pregnancy (Yes, No); dietary diversity (inadequate, adequate)	Ethnicity: Madhesi vs. Terai Dalit/Janajati/Muslim (aOR: 2.34; 95% CI 1.06–5.17); educational status: secondary level and higher vs. below secondary level (aOR: 2.87; 95% CI 1.52–5.45); frequency of antenatal visit: ≥ 4 times vs. < 4 times (aOR: 2.28; 95% CI 1.23–4.22); consumption of deworming medicine: Yes vs. No (aOR: 3.03; 95% CI 1.20–7.65); dietary diversity: adequate vs inadequate (aOR: 4.25; 95% CI 1.81–9.98)
Tripathi et al. (2020) [29]; underweight/normal vs. overweight/obesity	Age (< 30, 30–39, 40–49 years); education level (primary or below, secondary level, and bachelor and above); occupation (not employed outside home, business, public/private job, and others); vegetable/fruit consumption per week (≤ 21 servings and ≥ 22 servings); frequency of fast food consumption (occasional, once/twice a week, and three/more times a week); level of physical activity (low and moderate, high); parity (null parity, 1 parity, and multiparty); ever used any contraceptive devices (Yes, No)	Age: < 30 vs. 30–39 years (aOR: 13.855, 95% CI: 5.77–50.80), < 30 vs. 40–49 years (aOR: 18.79, 95% CI: 4.01–82.96); occupation: not employed vs. business (aOR: 7.39, 95% CI: 2.25–14.17); vegetables/fruits consumption per week: ≤ 21 servings vs. ≥ 22 servings (aOR: 0.14, (95% CI: 0,05–0.35); level of physical activity: high vs. low/moderate (aOR: 2.84, 95% CI: 1.18–6.83); parity: nulliparity vs. multiparity (aOR: 17.80, 95% CI: 4.04–89.06)
Sharma et al. (2020) [30]; anemia vs. nonanemia	Age (< 20 years, > 20 years); ethnicity (Dalit, disadvantage Janajati, and upper caste group); parity (0, 1, 2+)	Ethnicity: upper caste group vs. disadvantaged Janajati (aOR: 4.61, 95% CI 1.48–14.35)
Shah et al. (2020) [32]; BMI normal vs. overweight/obese	Monthly income (< 30,000, ≥ 30,000); wheat intake (regular, frequent, rare, and never); fast food intake (regular, frequent, rare, and never); dairy products intake (regular, frequent, rare, and never); fat intake (adequate, inadequate, and high); meat intake (regular, frequent, rare, and never)	Monthly income (*p* < 0.001); wheat intake (*p*=0.031); fast food intake (*p*=0.001); dairy products intake (*p*=0.046); fat intake (*p* < 0.001)
Bhattarai et al. (2018) [33]; (i) non-overweight/non-obese vs. overweight/obesity, (ii) abdominal obesity vs. nonabdominal obesity	Age (< 30 years, 30–40 years, 41–49 years); family size (< 5, > 5); marital status (unmarried/separated, married); alcoholic (Yes, No); protein intake (adequate, inadequate); parity (0, 1–2, ≥ 3); salt intake per day (> 5 g, < 5 g); eating outside once a day (Yes, No); contraceptive use (Yes, No)	*Factors associated with overweight/obesity:* age (*p*=0.001); family size (*p*=0.027); marital status (*p*=0.004); alcoholic (*p*=0.031); protein intake (*p*=0.002); parity (*p*=0.019) *factors associated with abdominal obesity:* age (*p* < 0.001); marital status (*p* < 0.001); parity (*p*=0.017); eating outside once a day (*p*=0.012); protein intake (*p*=0.002); contraceptive use (*p*=0.018)
Acharya et al. (2017) [9]; poor nutritional status (underweight + overweight/obese) vs. good nutritional status	Education level (illiterate, literate, primary, secondary, above secondary); material status (married, unmarried, and others); types of family (nuclear and joint); caste (Dalit, upper caste); Illness (Yes, No); food adequacy (Yes, No)	Education level (*p*=0.006); marital status (*p*=0.008); type of family (*p*=0.001); caste (*p*=0.001); illness (*p*=0.001); food adequacy (*p*=0.001)
Kanodia et al. (2016) [34]; anemia vs. nonanemia	Diet consumed (vegetarian, nonvegetarian); passage of worms (Yes, No); menarcheal status (premenarcheal, postmenarcheal); nutritional status (undernourished, normal, overweight); parental education (no formal schooling, primary level, and above)	Menarcheal status (*p*=0.031); nutritional status (*p*=0.023); parental education (*p*=0.015)
Bhandari et al. (2016) [35]; (i) normal vs. malnourished (BMI < 18.5 + BMI ≥ 25), (ii) normal vs. anemic	Educational status (illiterate, informal education, formal education); age (15–24, 25–34, 35–49 years); employment (unemployed, formal employment, agriculture, manual work); ethnicity (Dalit, disadvantaged Janajati (dJ), disadvantaged non-Dalit Terai caste (dnDT), religious minorities (Rm), relatively advantaged Janajati (aJ), upper caste); ecological region (mountain, hill, Terai); nutrition education by any organization (Yes, No)	*Factors associated with malnourished:* education: formal vs. informal education (aOR: 0.07, 95% CI: 0.6–0.8); Age: 35–49 vs. 15–24 years (aOR: 2.7, 95% CI: 2.5–3.0), 35–49 vs. 25–34 years (aOR: 1.3, 95% CI: 1.2–1.4); employment: manual work vs. unemployed (aOR: 1.9, 95% CI: 1.5–2.2), manual work vs. agriculture (aOR: 1.6, 95% CI: 1.3–1.9); ethnicity: upper caste vs. Dalit (aOR: 0.8, 95% CI: 0.7–0.8), upper caste vs. dJ (aOR: 0.6, 95% CI: 0.5–0.6), upper caste vs. dnDT (aOR: 0.6, 95% CI: 0.5–0.7), upper caste vs. Rm (aOR: 0.4, 95% CI: 0.3–0.5), upper caste vs. aJ (aOR: 0.6, 95% CI: 0.5–0.6); ecological regions: Terai vs. mountain (aOR: 0.2, 95% CI: 0.1–0.2), Terai vs. hill (aOR: 0.4, 95% CI: 0.3–0.5); nutrition education by any organization: Yes vs. No (aOR: 1.1, 95% CI: 1.0–1.2). *factors associated with anemia.* education: formal vs. informal education (aOR: 0.07, 95% CI: 0.7–0.8, formal vs. illiterate (aOR: 0.7, 95% CI: 0.7–0.8); age: 35–49 vs. 15–24 years (aOR: 1.3, 95% CI: 1.0–1.3), 35–49 vs. 25–34 years (aOR: 1.1, 95% CI: 1.0–1.2); employment: manual work vs. unemployed (aOR: 1.6, 95% CI: 1.4–1.8), manual work vs. agriculture (aOR: 1.7, 95% CI: 1.5–2.0); ethnicity: upper caste vs. Dalit (aOR: 0.8, 95% CI: 0.7–0.9), upper caste vs. dJ (aOR: 0.9, 95% CI: 0.8–0.9), upper caste vs. dnDT (aOR: 0.5, 95% CI: 0.5–0.6), upper caste vs. Rm (aOR: 0.5, 95% CI: 0.4–0.6), upper caste vs. aJ (aOR: 0.6, 95% CI: 0.5–0.6); ecological regions: Terai vs. mountain (aOR: 0.05, 95% CI: 0.04–0.07); nutrition education by any organization: Yes vs. No (aOR: 1.1, 95% CI: 1.0–1.2)

**Table 4:** Summary matrix of predictors of malnutrition among women of reproductive age in Nepal.

Domain	Factor	Effect	Population	Effect size (if reported)	Reference
Demographic	Younger age (15–24 yrs)	↑ Underweight	WRA	aOR 2.7 (95% CI: 2.5–3.0)	Bhandari et al. 2016 [35]
	Middle age (30–39 yrs); Older age (≥ 40–49 yrs)	↑ Overweight/obesity	WRA	30–39 years (aOR: 13.855, 95% CI: 5.77–50.80); 40–49 years (aOR: 18.79, 95% CI: 4.015–82.96);	Tripathi et al. 2020 [29]
	Ethnicity (Janajati, Dalit, Muslim)	↑ Anemia	Pregnant women	Disadvantaged Janajati (aOR: 4.615, 95% CI 1.48–14.35), Terai Dalit/Janajati/Muslim (aOR: 2.34; 95% CI 1.06–5.17);	Sharma et al. 2020 [30]; Yadav et al. 2021 [28]
	Ethnicity (Dalit, disadvantaged Janajati (dJ), disadvantaged non-Dalit Terai caste (dnDT), religious minorities (Rm), relatively advantaged Janajati (aJ), upper caste)	↓ Malnutrition in upper caste compared with marginalized groups	WRA	Upper caste vs. Dalit (aOR: 0.8, 95% CI: 0.7–0.8), upper caste vs. dJ (aOR: 0.6, 95% CI: 0.5–0.6), upper caste vs. dnDT (aOR: 0.6, 95% CI: 0.5–0.7), upper caste vs. Rm (aOR: 0.4, 95% CI: 0.3–0.5), upper caste vs. aJ (aOR: 0.6, 95% CI: 0.5–0.6)	Bhandari et al. 2016 [35]
	Geographic region (Terai vs. hill vs. mountain)	↑ Malnutrition in hill/mountain compared with Terai	WRA	Terai vs. mountain (aOR: 0.2, 95% CI: 0.1–0.2), Terai vs. hill (aOR: 0.4, 95% CI: 0.3–0.5)	Bhandari et al. 2016 [35]
		↑ Anemia in hill/mountain compared with Terai		Terai vs. mountain (aOR: 0.05, 95% CI: 0.04–0.07)	

Socioeconomic	Higher education	↓ Malnutrition and Anemia	WRA	Formal vs. informal education (aOR: 0.07, 95% CI: 0.6–0.8); below secondary level (aOR: 2.87; 95% CI 1.52–5.45)	Bhandari et al. 2016 [35]; Yadav et al. 2021 [28]
	Occupation	↑ Overweight/obesity	WRA	Not employed vs. business (aOR: 7.39, 95% CI: 2.25–14.17)	Tripathi et al. 2020 [29]
		↑ Undernutrition	Adolescents and WRA	Employed for cash vs. employed not for cash/unemployed (aOR: 6.61, 95% CI: 2.17–20.12)	Aryal et al. 2022 [27]
	Household food insecurity	↑ Thinness	Female (15–19) years	Household food insecurity (*p*=0.002)	Giri et al.2023 [24]

Dietary	Low dietary diversity (≤ 4 food groups)	↑ Undernutrition and Anemia	Adolescents/Pregnant women	> 5 food groups vs. ≤ 4 food groups (aOR: 10.06, 95% CI: 3.35–30.27).1	Aryal et al. 2022 [27]; Yadav et al. 2021 [28]
	Fat intake (adequate, inadequate, high); dairy products intake (regular, frequent, rare, never)	↑ Overweight/obesity	WRA	Fat intake (*p* < 0.001); dairy products intake (*p*=0.046)	Shah et al. 2020 [32]
	Low protein intake; alcohol consumption	↑ Overweight/obesity and abdominal obesity	WRA	Protein intake (*p*=0.002); alcoholic (*p*=0.031)	Bhattarai et al. 2018 [33]

Reproductive	Early marriage/pregnancy (< 20 yrs)	↑ Malnutrition	WRA	Age at first pregnancy (*p*=0.006); parity (*p*=0.004); number of children (*p* < 0.001)	Paudel et al. 2023 [22]
	Higher parity (≥ 3 children)	↑ Malnutrition	WRA	Nulliparity vs. multiparity (aOR: 17.80, 95% CI: 4.04–89.06)	Tripathi et al. 2020 [29], Paudel et al. 2023 [22]
	Fewer antenatal visits (< 4)	↑ Anemia	Pregnant women	Frequency of antenatal visit: ≥ 4 times vs. < 4 times (aOR: 2.28; 95% CI 1.23–4.22);	Yadav 2021 [28]

Physical activity	Low physical activity	↑ Overweight/obesity		Level of physical activity: high vs. low/moderate (aOR: 2.84, 95% CI: 1.18–6.83)	Tripathi et al. 2020 [29]

Deworming	No deworming medication	↑ Anemia	Pregnant women	Consumption of deworming medicine: Yes vs. No (aOR: 3.03; 95% CI 1.20–7.65);	Yadav et al. 2021 [28]

## Data Availability

The data are available from the corresponding author upon request.

## Supporting Information

The detailed search strategy adopted for this scoping review, including complete search terms and database-specific strategies applied in PubMed, DOAJ, CINAHL, NepJOL, and additional manual and gray literature searches, is provided in Supporting File 1.
