# Supplementary material for: Prevalence and Predictors of Obesity, Undernutrition, and Anemia in Women of Reproductive Age Living in Nepal: A Scoping Review
Source: J Nutr Metab. 2025 Oct 28;2025:8897249. doi: 10.1155/jnme/8897249 (PMC12585867; doi:10.1155/jnme/8897249)
Supplement: Supporting Information — Additional supporting information can be found online in the Supporting Information section. [file 8897249.f1.docx]

**Search Strategy for the Scoping Review: Prevalence and Predictors of Obesity, Undernutrition, and Anemia in Women of Reproductive Age Living in Nepal**

Date of final search: June 15, 2024

## 1. PubMed

("Nutritional Disorder"[Mesh] OR "Nutritional Status"[Mesh] OR malnutrition[tiab] OR undernutrition[tiab] OR undernourished[tiab] OR thinness[tiab] OR "body mass index"[tiab] OR BMI[tiab]) AND ("Women of Reproductive Age"[Mesh] OR WRA[tiab] OR adolescent girls[tiab] OR women[tiab] OR female[tiab]) AND Nepal[tiab])

(overweight[tiab] OR obesity[tiab] OR "central obesity"[tiab] OR overnutrition[tiab] OR "body mass index"[tiab] OR BMI[tiab]) AND ("Women of Reproductive Age"[Mesh] OR WRA[tiab] OR adolescent girls[tiab] OR women[tiab] OR female[tiab]) AND Nepal[tiab])

(anemia[tiab] OR anaemia[tiab] OR hemoglobin[tiab] OR haemoglobin[tiab]) AND ("Women of Reproductive Age"[Mesh] OR WRA[tiab] OR adolescent girls[tiab] OR women[tiab] OR female[tiab]) AND Nepal[tiab])

(MUAC[tiab] OR "Mid upper arm circumference"[tiab]) AND ("Women of Reproductive Age"[Mesh] OR WRA[tiab] OR adolescent girls[tiab] OR women[tiab] OR female[tiab]) AND Nepal[tiab])

[Mesh] = Medical Subject Headings (controlled vocabulary).

[tiab] = Title and Abstract fields (free-text search).

## 2. Directory of Open Access Journals (DOAJ)

(malnutrition OR undernutrition OR overweight OR obesity OR anemia OR "body mass index" OR BMI OR MUAC) AND (women OR female OR adolescent OR reproductive) AND
(Nepal)

## 3. CINAHL (via EBSCOhost)

("Nutritional Disorder" OR malnutrition OR undernutrition OR thinness OR overweight OR obesity OR "body mass index" OR BMI OR MUAC OR anemia OR anaemia OR hemoglobin OR haemoglobin) AND ("Women of Reproductive Age" OR WRA OR adolescent OR female OR women) AND (Nepal)

## 4. NepJOL (Nepal Journals Online)

(malnutrition OR undernutrition OR overweight OR obesity OR BMI OR anemia OR hemoglobin OR MUAC) AND (women OR reproductive OR adolescent)

## 5. Manual and Grey Literature Search

- Reference list screening of all included full-text articles.
- Hand-searching key Nepalese journals not indexed in international databases.
- Google Scholar search using simplified terms: malnutrition AND women AND Nepal
- Date range: January 1, 2000 – June 15, 2024

## 6. Japanese University Library Databases

- Additional searches were conducted through subscription-based databases accessed via Japanese university libraries (Osaka University and Kansai Medical University Libraries) by Sakai (SH) to supplement the above databases and identify relevant studies not indexed in international or open-access databases.

All the articles were exported and handled at ENDNOTE 20 reference management software
